# Inhibition of Proteasomal Degradation of Rpn4 Impairs Nonhomologous End-Joining Repair of DNA Double-Strand Breaks

**DOI:** 10.1371/journal.pone.0009877

**Published:** 2010-04-01

**Authors:** Donghong Ju, Xiaogang Wang, Seung-Wook Ha, Jiejun Fu, Youming Xie

**Affiliations:** 1 Barbara Ann Karmanos Cancer Institute, Wayne State University School of Medicine, Detroit, Michigan, United States of America; 2 Department of Pathology, Wayne State University School of Medicine, Detroit, Michigan, United States of America; Karolinska Institutet, Sweden

## Abstract

**Background:**

The proteasome homeostasis in *Saccharomyces cerevisiae* is regulated by a negative feedback circuit in which the transcription factor Rpn4 induces the proteasome genes and is rapidly degraded by the assembled proteasome. The integrity of the Rpn4-proteasome feedback loop is critical for cell viability under stressed conditions. We have demonstrated that inhibition of Rpn4 degradation sensitizes cells to DNA damage, particularly in response to high doses of DNA damaging agents. The underlying mechanism, however, remains unclear.

**Methodology/Principal Findings:**

Using yeast genetics and biochemical approach we show that inhibition of Rpn4 degradation displays a synthetic growth defect with deletion of the *MEC1* checkpoint gene and sensitizes several checkpoint mutants to DNA damage. In addition, inhibition of Rpn4 degradation leads to a defect in repair of double-strand breaks (DSBs) by nonhomologous end-joining (NHEJ). The expression levels of several key NHEJ genes are downregulated and the recruitment of Yku70 to a DSB is reduced by inhibition of Rpn4 degradation. We find that Rpn4 and the proteasome are recruited to a DSB, suggesting their direct participation in NHEJ. Inhibition of Rpn4 degradation may result in a concomitant delay of release of Rpn4 and the proteasome from a DSB.

**Conclusion/Significance:**

This study provides the first evidence for the role of proteasomal degradation of Rpn4 in NHEJ.

## Introduction

The *S. cerevisiae RPN4* gene (also named *SON1* and *UFD5*) was originally isolated as a suppressor of *sec63-101*, a temperature-sensitive mutant of *SEC63*, which encodes an essential component of the endoplasmic reticulum translocation channel [1], [2]. Subsequent work showed that deletion of *RPN4* inhibits the degradation of several model substrates of the N-end rule and UFD (*U*b *f*usion *d*egradation) pathways, suggesting the involvement of Rpn4 in proteasomal degradation [3]. The exact functional role of Rpn4 in protein degradation, however, remained unclear until recent studies revealed that Rpn4 is a transcription factor for the proteasome genes [4], [5]. This finding explains why the proteasome activity is diminished in an *rpn4Δ* mutant. Interestingly, Rpn4 is an extremely short-lived protein (t_1/2_≤2 min) and degraded by the proteasome [5]–[10]. Moreover, stabilization of Rpn4 by inhibition of the proteasome activity leads to an increase in the expression levels of the proteasome genes [11], [12]. Together, these observations led to a model in which the proteasome homeostasis is regulated by a negative feedback circuit. On the one hand, Rpn4 upregulates the proteasome genes; on the other hand, Rpn4 is rapidly degraded by the assembled/active proteasome. The Rpn4-proteasome negative feedback circuit provides an efficient and sensitive means to control the in vivo proteasome abundance. The proteasome genes in higher eukaryotes including humans are regulated by a similar negative feedback mechanism even though the homologs of Rpn4 have not yet been identified [13]–[16].

In addition to the proteasome genes, Rpn4 appears to influence the expression of a large number of other genes involved in protein ubiquitylation, DNA repair and other cellular processes [4], [17]–[23]. Interestingly, the promoter of *RPN4* carries the binding sites for heat-shock transcription factor (Hsf1), multidrug resistance-related transcription factors (Pdr1 and Pdr3), and Yap1, a transcription factor that plays an important role in response to oxidation and DNA damage [18], [24], [25]. These transcription factors are activated by a variety of environmental stressors and in turn induce *RPN4* expression [11], [12], [17], [18], [24]–[26]. These observations suggest that Rpn4 may serve as a major stress- responsive mediator. The Rpn4-proteasome negative feedback loop likely plays a central role in the Rpn4-mediated stress response network, not only by maintaining the proteasome homeostasis but also by gauging the expression levels of other Rpn4 target genes through proteasomal degradation of Rpn4. In support of this hypothesis, our recent studies demonstrated that disruption of each of the two branches of the Rpn4-proteasome negative feedback loop, namely Rpn4-induced proteasome expression and proteasomal degradation of Rpn4, severely reduces cell viability under stressed conditions [27], [28].

Rpn4 can be degraded by two distinct mechanisms, ubiquitin (Ub)-dependent and -independent [6]. Our recent studies showed that the N-terminal 10 amino acids are required for the Ub-independent degradation of Rpn4, whereas residues 211–229 constitute the Ub-dependent degradation signal [6]–[10]. Simultaneous deletions of residues 1–10 and 211–229 substantially stabilize Rpn4, and yet, do not impair its transcriptional activity [28], [29]. Taking advantage of this stabilized Rpn4 mutant (Rpn4_Δ1–10/Δ211–229_, referred to as Rpn4* for abbreviation), we demonstrated that inhibition of Rpn4 degradation causes cell hypersensitivity to DNA damage, particularly in response to high doses of DNA damaging agents [28]. It is possible that expression of Rpn4* may affect checkpoint activation in response to DNA damage. Alternatively, it may lead to a defect in DNA repair.

In this study we sought to understand how inhibition of Rpn4 degradation sensitizes cells to DNA damage. We found that expression of Rpn4*, while imposing no effect on DNA checkpoint activation, displays a synthetic growth defect with deletion of the *MEC1* checkpoint gene and sensitizes several checkpoint mutants to DNA damage. We further demonstrated that expression of Rpn4* impairs NHEJ but not homologous recombination (HR) repair of DSBs. The expression levels of several key NHEJ genes are downregulated and the recruitment of Yku70 to a DSB is reduced in the cells expressing Rpn4*. Interestingly, Rpn4 is recruited to a DSB and inhibition of Rpn4 degradation may cause a concomitant delay of the dissociation of Rpn4 and the proteasome from the DSB. These observations suggest that inhibition of Rpn4 degradation may affect NHEJ through different mechanisms.

## Results

### Inhibition of Rpn4 degradation does not affect checkpoint activation

**Figure 1:**
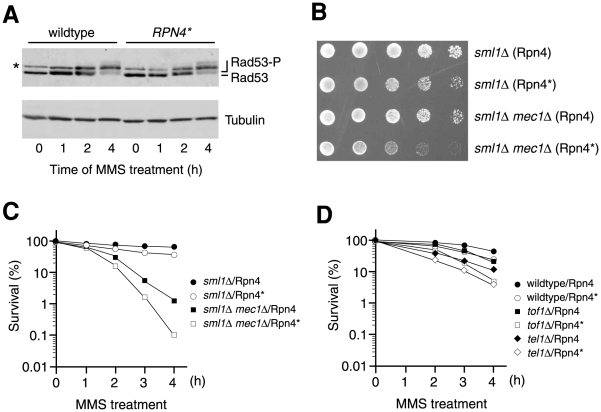
Synergistic effects caused by inhibition of Rpn4 degradation and checkpoint mutations. (A) Inhibition of Rpn4 degradation does not affect Rad53 phosphorylation. Cells expressing C-terminally FLAG-tagged Rad53 with either Rpn4 or Rpn4* were treated with 0.1% MMS and cell extracts were prepared at different time points and subjected to SDS-PAGE (6% gel), followed by immunoblotting with an anti-FLAG antibody (upper panel). The membrane was reprobed with an anti-α tubulin antibody to compare the loading (lower panel). Phosphorylated and unphosphorylated species of Rad53 were marked. An asterisk indicated a cross-reactive band with the anti-FLAG antibody. (B) A synthetic growth defect caused by inhibition of Rpn4 degradation and deletion of *MEC1*. The growth of *sml1Δ* and *sml1Δ mec1Δ* cells expressing either Rpn4 or Rpn4* was assessed by a five-fold serial dilution assay. (C, D) Inhibition of Rpn4 degradation sensitizes checkpoint mutants *mec1Δ* (C), *tof1Δ* and *tel1Δ* (D) to MMS. The colony formation assay was used to measure the survival rates, which are expressed as a percentage of the value of untreated cells set at 100%. The data shown are the mean of at least three independent experiments with deviation less than 10%.

To examine whether expression of Rpn4* could affect DNA checkpoint activation, we compared the phosphorylation of Rad53 in response to MMS between the cells expressing wildtype Rpn4 or Rpn4*. Rad53 is a central transducer of DNA damage responses in *S. cerevisiae* and its phosphorylation is a hallmark of checkpoint activation [30], [31]. A plasmid encoding C-terminally FLAG-tagged Rad53 was co-transformed with low-copy vectors expressing *RPN4* or RPN4* from the native *RPN4* promoter into an *rpn4Δ* strain. The transformants were treated with MMS and cell extracts were prepared at different time points. The phosphorylated and unphosphorylated Rad53 species were separated by SDS-PAGE and detected by immunoblotting analysis with an anti-FLAG antibody. As shown in Figure 1A, the kinetics of Rad53 phosphorylation in response to the MMS treatment was similar between the cells expressing *RPN4* or RPN4*. Thus, inhibition of Rpn4 degradation does not impair checkpoint activation in response to DNA damage.

### Synergistic effects caused by inhibition of Rpn4 degradation and checkpoint mutations

We then wondered if inhibition of Rpn4 degradation could display a synthetic growth defect with DNA checkpoint mutations. We decided to measure the synthetic effect of deletion of *MEC1* and expression of Rpn4* since Mec1 is the major checkpoint protein in *S. cerevisiae* [19], [31]–[33]. Low-copy plasmids expressing *RPN4* or RPN4* were transformed into a *mec1Δ* mutant and an isogenic *MEC1* strain. Note that the *SML1* gene has been deleted in these two strains to suppress the lethality of single deletion of *MEC1* [30]. Whereas Rpn4* showed only a mild effect on the *sml1Δ* control strain, it markedly slowed the growth of the *mec1Δ sml1Δ* mutant (Figure 1B). This analysis demonstrated that inhibition of Rpn4 degradation and a defect in DNA checkpoint activation can cause a synthetic negative effect on cell growth.

We went on to examine whether expression of Rpn4* could exacerbate the sensitivity of checkpoint mutants to DNA damaging agents. To this end, we transformed the RPN4* and *RPN4* plasmids into the *mec1Δ sml1Δ mutant*. The isogenic *sml1Δ* strain was used as a wildtype control. The transformants were treated with 0.01% MMS for 0, 1, 2, 3 and 4 h, and the survival rates were measured by the colony formation assay. Although Rpn4* only had a marginal effect on the wildtype strain under this low dose MMS stress, it reduced the survival rate of the *mec1Δ* mutant by ∼10 fold after 4 h treatment (Figure 1C). We also tested the effect of Rpn4* on two other checkpoint mutants, *tof1Δ* and *tel1Δ*, using a similar assay except that the MMS concentration was increased to 0.03%. Tof1 is a subunit of the Tof1-Mrc1-Csm3 checkpoint complex, which acts at the stalled replication fork [34]–[36]. Tel1 is the yeast homolog of the human ataxia telangiectasia (ATM) checkpoint protein, primarily involved in telomere length regulation [37], [38]. As shown in Figure 1D, Rpn4* enhanced the sensitivity of *tof1Δ* and *tel1Δ* mutants to MMS. Thus, expression of Rpn4*, while not directly affecting checkpoint activation, reduces the viability of checkpoint mutants in response to DNA damage, suggesting that inhibition of Rpn4 degradation may affect DNA repair.

### Inhibition of Rpn4 degradation impedes DSB repair by NHEJ

**Figure 2:**
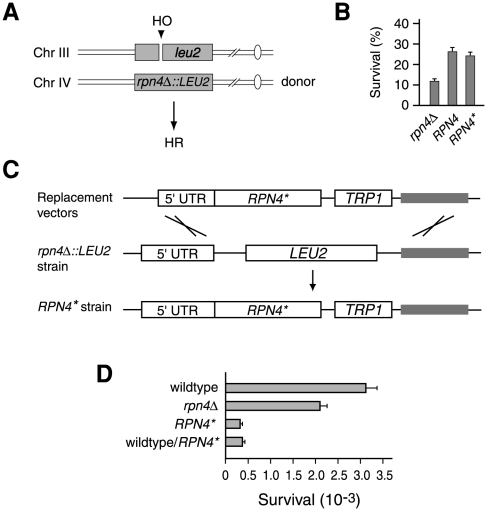
Inhibition of Rpn4 degradation leads to a defect in NHEJ. (A) An experimental system to measure HR. The *RPN4* ORF on chromosome IV of the YFP17 strain was replaced with a *LEU2* selection marker, which also serves as a homologous template for HR repair of the HO-cut DSB at *leu2* on chromosome III. (B) Rpn4* has no significant effect on HR. Low-copy vectors expressing *RPN4* or RPN4* from the native *RPN4* promoter or a void vector were transformed into the *rpn4Δ::LEU2* strain in (A). The resulting transformants were spread on galactose and glucose plates. Survival rates were expressed as the ratios of colony counts on galactose plates to those on glucose plates. (C) Construction of an RPN4* strain. The *rpn4Δ::LEU2* allele of the *rpn4Δ::LEU2* strain was replaced with an RPN4*-3ha::TRP1 cassette via site-specific recombination (see Materials and Methods). 5′-UTR represents the *RPN4* promoter region whereas the boxes in gray are the homologous sequences between the pRS304 and pRS305 backbones. (D) Rpn4* impedes NHEJ. Wildtype, RPN4* and *rpn4Δ* strains and a wildtype transformant with an exogenous RPN4* plasmid were spread on galactose and glucose plates. Survival rates were calculated by dividing the colony numbers on galactose plates by those on glucose plates. Data represented are the mean of three independent experiments.

Our early study showed that the effect of Rpn4* on cell viability became increasingly prominent when cells were treated with high doses of DNA damaging agents [28], suggesting that degradation of Rpn4 may be important for repairing severely damaged DNA, i.e. DSBs. To test this conjecture, we decided to examine whether expression of Rpn4* leads to a defect in either HR or NHEJ repair of a single DSB induced by the HO endonuclease. Specifically, we used the haploid yeast strain YFP17, which expresses the HO endonuclease from the *GAL10* promoter and carries an HO recognition site in the *leu2* locus on chromosome III [39]. The native HO cut sites at the *MAT*, *HML*, and *HMR* loci have been deleted in this strain. Therefore, a single DSB is created at the *leu2* locus in this donor-less strain upon HO induction by galactose. To measure the effect of Rpn4* on HR efficiency, we replaced the *RPN4* open reading frame (ORF) on chromosome IV with a *LEU2* selection marker, which can serve as a homologous template for HR repair of the DSB at the *leu2* locus (Figure 2A). Low-copy vectors expressing RPN4* or *RPN4* from the native *RPN4* promoter or a void vector were transformed into the *rpn4Δ::LEU2* strain. The relative survival rates of the transformants on galactose versus glucose plates, which reflect the efficiency of DSB repair by HR, were measured by the colony formation assay. As shown in Figure 2B, the RPN4* strain had a similar relative survival rate as the wildtype *RPN4* strain, suggesting that inhibition of Rpn4 degradation has no significant effect on HR. In contrast, the relative survival rate of the *rpn4Δ* strain was only ∼50% of that of the wildtype strain, indicating that Rpn4 is important for HR. In line with this observation, we found that it was generally less efficient to generate site-specific recombinant strains from an *rpn4Δ* background strain than from its wildtype counterpart (data not shown).

We then examined the effect of Rpn4* on NHEJ. An RPN4* strain was constructed by replacing the *rpn4Δ::LEU2* allele with an RPN4*::TRP1 cassette via site-specific recombination (Figure 2C). A triple ha tag (3ha) was added to the C-terminus of Rpn4* for immunoprecipitation analysis with an anti-ha antibody. To create a control strain, we inserted a C-terminal 3ha tag and a *TRP1* selection marker at the *RPN4* locus in the parental YFP17 strain. An *rpn4Δ::TRP1* strain was also generated by replacing the *RPN4* ORF of YFP17 with a *TRP1* selection marker to assess the requirement of Rpn4 in NHEJ. The relative survival rates of these three strains on galactose versus glucose plates were measured by the colony formation assay. Because of the lack of HR template in these strains, growth on galactose plates depends on NHEJ. As shown in Figure 2D, the survival rate of RPN4* cells was 6–7 fold lower than that of their wildtype counterparts. Introduction of an exogenous RPN4* plasmid into the wildtype cells also dramatically reduced their viability on galactose plates (Figure 2D). These results imply that inhibition of Rpn4 degradation may impair NHEJ. Deletion of *RPN4* also affected NHEJ, but to a much smaller extent compared to expression of Rpn4* (Figure 2D).

**Figure 3:**
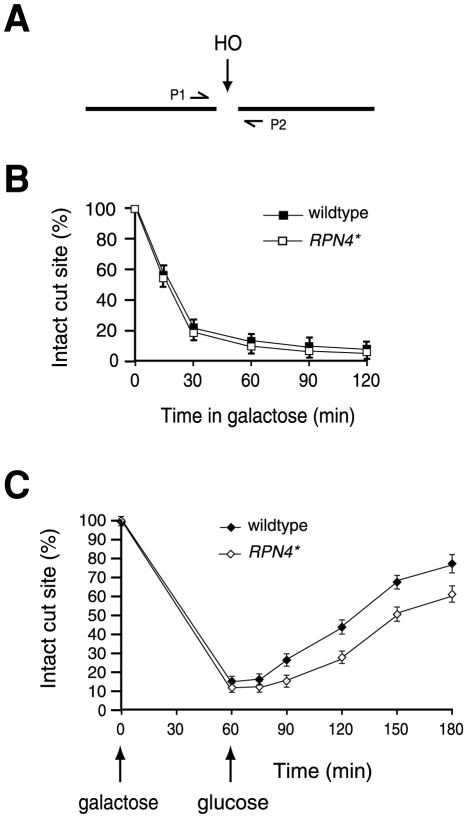
The kinetics of NHEJ is slower in the RPN4* strain. (A) Positions of the primers (P1 and P2) used for DSB analysis. (B) Measurement of DSB induction. Genomic DNA was prepared from wildtype and RPN4* strains at different time points of galactose induction and analyzed by qRT-PCR. The extent of DSB induction was determined by the ratio of PCR product from primers P1 and P2 to that from a pair of control primers amplifying an unrelated gene (*PRE1*). (C) Slower NHEJ in the RPN4* strain. Wildtype and RPN4* cells were induced by galactose for 1 h, followed by transfer to glucose medium to terminate HO expression. Genomic DNA was prepared at different time points as indicated, and subjected to qRT-PCR analysis as (B).

To further examine the effect of Rpn4* on NHEJ, we decided to compare the kinetics of DSB repair by NHEJ between the wildtype and RPN4* strains. We first monitored the appearance of HO-induced DSB. Specifically, genomic DNA was prepared from cell cultures withdrawn at different time points after galactose induction and analyzed by quantitative real-time PCR (qRT-PCR) using a pair of primers (P1 and P2) flanking the HO cut site (Figure 3A). A decrease in the yield of PCR product is expected after the chromosome is broken. Another pair of primers amplifying an unrelated gene was used as a control to normalize the qRT-PCR results. We found that the kinetics of DSB formation was similar between the wildtype and RPN4* strains (Figure 3B). The percentage of cells with a DSB was slightly but reproducibly higher in the RPN4* strain than in its wildtype counterpart. This difference likely reflects more efficient early repair (in competition with HO cutting) in the wildtype strain relative to the RPN4* strain. Since more than 80% of cells have acquired a DSB after 60 min of galactose induction, we decided to terminate HO expression at 60 min by transferring the cell cultures into glucose medium to follow DSB repair. Chromatin fractions were prepared at different time points and subjected to qRT-PCR analysis with primers P1 and P2. As shown in Figure 3C, the wildtype cells repaired the DSB more efficiently than their RPN4* counterparts after termination of HO expression. Together, these results demonstrate that inhibition of Rpn4 degradation impairs NHEJ.

### Altered expression of NHEJ genes in the RPN4* strain

**Figure 4:**
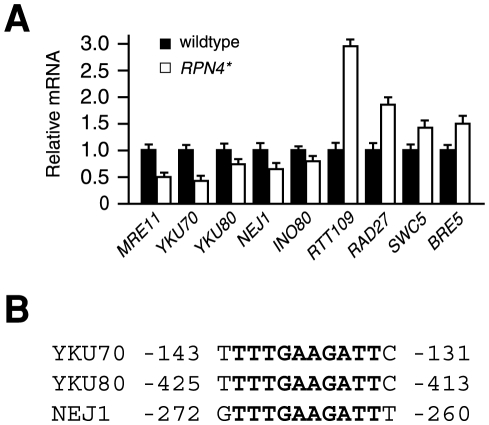
Altered expression of NHEJ genes by inhibition of Rpn4 degradation. (A) The expression levels of NHEJ genes in wildtype and RPN4* strains were analyzed by qRT-PCR. *ACT1* was used as an internal control. Values were presented as relative ratios to the mRNA levels in the wildtype strain. Results shown here are the mean of three independent experiments. (B) A 10-bp consensus sequence (highlighted in bold) exists in the promoters of *YKU70*, *YKU80*, and *NEJ1*.

To gain insights into the NHEJ defect caused by inhibition of Rpn4 degradation, we compared the expression levels of all the genes known to be related to NHEJ between the wildtype and RPN4* strains by qRT-PCR analysis. Forty-one genes were analyzed (see Table S1), including the structural/catalytic NHEJ components (*MRE11*, *RAD50*, *XRS2*, *YKU70*, *YKU80*, *DNL4*, *LIF1*, and *NEJ1*) and those whose mutations lead to partial defects in NHEJ [40]–[49]. We found that the expression levels of *MRE11*, *YKU70*, *YKU80*, *NEJ1*, and *INO80* were lower in the RPN4* strain than in the wildtype strain (Figure 4A). In contrast, *RTT109*, *RAD27*, *SWC5*, and *BRE5* were expressed at a higher level in the RPN4* strain than in the wildtype strain. The expression levels of other NHEJ genes were comparable between these two strains (data not shown). Interestingly, there is a 10-bp consensus sequence in the promoters of *YKU70*, *YKU80*, and *NEJ1* (Figure 4B), suggesting that these three genes may be coordinately downregulated in the presence of Rpn4*.

### Reduced recruitment of Yku70 to a DSB in the RPN4* strain

**Figure 5:**
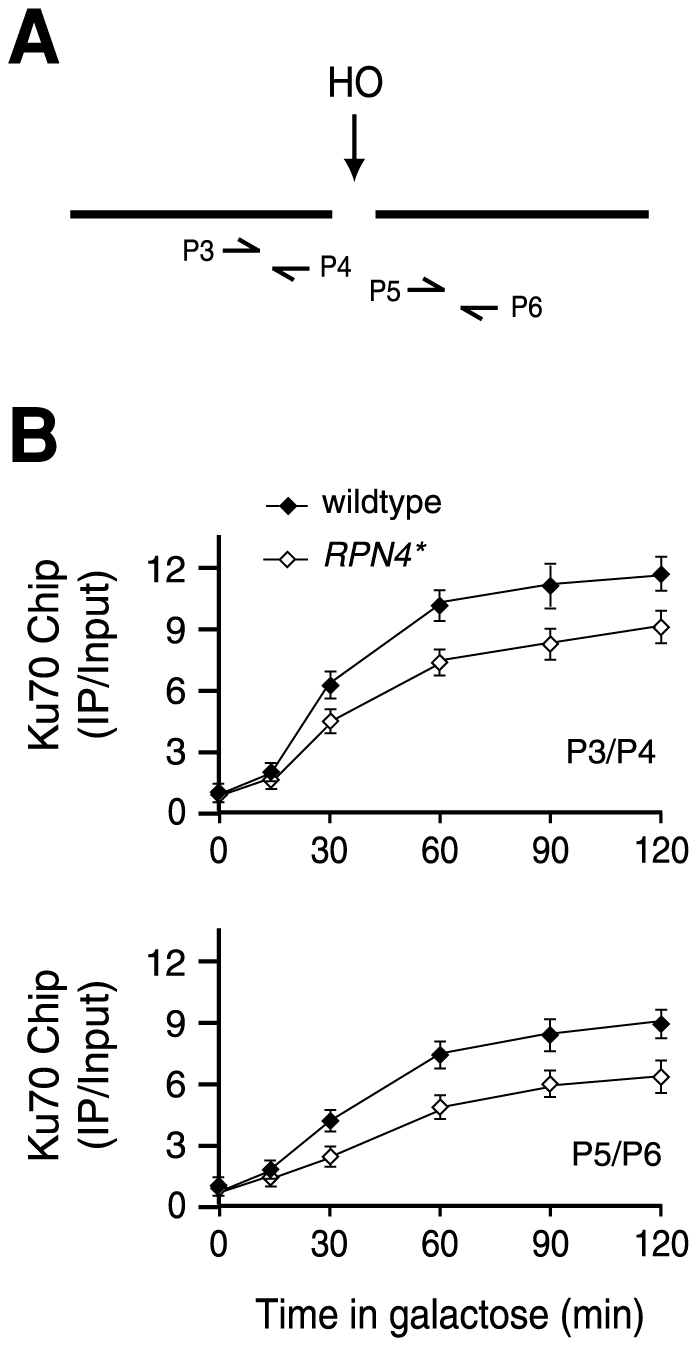
Inhibition of Rpn4 degradation reduces the recruitment of Yku70 to a DSB. (A) Two sets of primers, proximal (P3/P4) and distal (P5/P6) to the HO cut site, were used in the ChIP assay. (B) The kinetics of Yku70 recruitment to a DSB in the wildtype and RPN4* strains. Cell extracts were prepared from samples collected at different time points after galactose induction and subjected to ChIP as described in Materials and Methods. Specific binding of Yku70 to the DSB was expressed as a ratio of the PCR product from immunoprecipitated DNA to input DNA, and normalized against the nonspecific ChIP signal obtained from the sample before HO induction (time zero), which was set at 1.0. Both ChIP results using the proximal (upper panel) and distal (lower panel) sets of primers were shown. The values are the mean of three independent experiments.

The downregulation of *YKU70* and *YKU80* in the RPN4* strain prompted us to examine if the recruitment of the Ku complex to a DSB is affected by expression of Rpn4*. To this end, we attached a triple FLAG tag (3FLAG) to the C-terminus of Yku70 expressed from its chromosomal locus in the wildtype and RPN4* strains. Note that addition of the 3FLAG tag to Yku70 did not affect the cell's NHEJ efficiency (data not shown). The binding of Yku70 to the DSB was determined by the chromatin immunoprecipitation (ChIP) assay. Based on the kinetics of DSB formation in galactose medium (Figure 3B), we collected chromatin samples co-immunoprecipitated with Yku70 by an anti-FLAG antibody at different time points. Two pairs of primers were used for qRT-PCR to quantitate the recovered DNA by ChIP, with one corresponding to the proximal side and the other on the distal side of the HO cut site (Figure 5A). We found that Yku70 was recruited immediately following the formation of DSB in both wildtype and RPN4* strains, peaking at ∼90 min (Figure 5B). However, the initial recruitment of Yku70, i.e. at 30 min, was apparently slower in the RPN4* strain compared to that in the wildtype strain. In addition, the ChIP signals were generally weaker in the RPN4* strain than in the wildtype strain. The reduced recruitment of Yku70 to the DSB is in line with the downregulation of *YKU70* in the RPN4* strain.

### Concomitantly delayed dissociation of Rpn4* and the proteasome from a DSB

**Figure 6:**
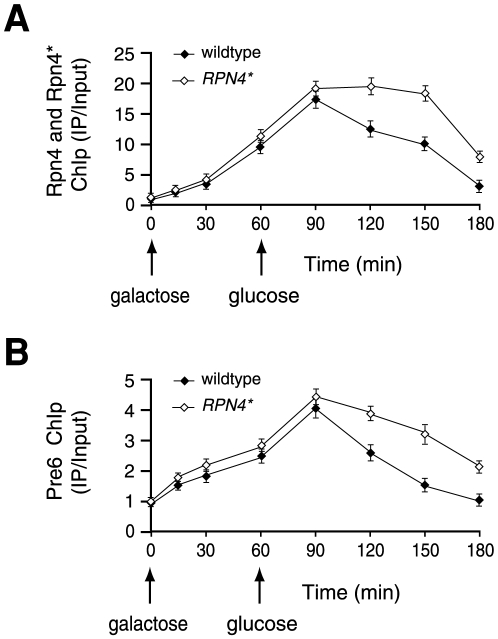
Delayed release of Rpn4* and the proteasome from a DSB in the RPN4* strain. Wildtype and RPN4* strains in which a 3FLAG tag was added to the C-terminus of Pre6 were induced by galactose for 1 h, and then transferred to glucose medium to terminate HO expression. Samples were prepared at different time points for ChIP analysis with an anti-ha antibody for Rpn4-3ha and Rpn4*-3ha (A) or an anti-FLAG antibody for Pre6-3FLAG (B). The binding of Rpn4, Rpn4* or Pre1 to the DSB was expressed as a ratio of the PCR product from immunoprecipitated DNA to input DNA, and normalized against the nonspecific ChIP signal obtained from the sample at time zero (before HO induction), which was set at 1.0. Shown here are the qRT-PCR results with primers P3 and P4. Data are presented as the mean of three independent experiments. Similar results were obtained using primers P5 and P6 (data not shown).

Whereas the effect of Rpn4* on NHEJ may be attributed to the deregulation of some NHEJ genes, we suspected that Rpn4 might affect NHEJ via other routes. This thought originated from the observations that Rpn4 interacts with the proteasome and that the proteasome directly participates in NHEJ [5], [42], [50]. We, therefore, examined if Rpn4 could be recruited to a DSB, and if so, is there any difference in the kinetics of recruitment and dissociation of Rpn4 and Rpn4* to and from the DSB. Taking advantage of the 3ha tag attached to the C-termini of Rpn4 and Rpn4* in the wildtype and RPN4* strains, we were able to use an anti-ha antibody to perform the ChIP assay. Cells were grown in galactose medium to induce DSB formation, followed by transfer to glucose medium to terminate HO expression. Aliquot samples were withdrawn at different time points during galactose induction and after termination of HO induction to measure the Rpn4 and Rpn4* ChIP signals by qRT-PCR. This assay showed that both Rpn4 and Rpn4* were recruited to the DSB (Figure 6A). Whereas a modestly larger amount of Rpn4* was recruited to the DSB than Rpn4, probably due to a higher steady-state level of Rpn4*, the kinetics of recruitment was quite similar between Rpn4 and Rpn4*, both peaking 30 min after HO shut-off (Figure 6A). Interestingly, the Rpn4 ChIP signal rapidly decreased after approaching the peak. In contrast, the maximal Rpn4* ChIP signal persisted for almost 60 min before it began to decline. These results suggest that inhibition of Rpn4 degradation may delay the dissociation of Rpn4 from the DSB.

We then tested if Rpn4* could affect the binding and dissociation of the proteasome to and from a DBS. For the ChIP analysis, a 3FLAG tag was added to the C-terminus of the Pre6 proteasome subunit expressed from its chromosomal locus. Although relatively stronger Pre6 ChIP signals were recorded in the RPN4* strain than in the wildtype strain, likely resulting from upregulation of Pre6 in the RPN4* strain [28], the kinetics of Pre6 recruitment was comparable in these two strains, reaching the peak 30 min after termination of HO induction (Figure 6B). Thus, Rpn4* does not appear to affect the initial binding of the proteasome to the DSB. This assay also suggested that Rpn4 and the proteasome may be recruited simultaneously to the DSB because the binding of Rpn4 and Pre6 to the DSB followed a similar kinetics. Interestingly, the dissociation of Pre6 from the DSB was apparently slower in the RPN4* strain than the wildtype strain (Figure 6B). Together, these results imply a concomitant delay in releasing Rpn4* and the proteasome from the DSB.

## Discussion

In this study we demonstrated that inhibition of Rpn4 degradation impairs NHEJ. Our data suggest that this detrimental effect may involve different mechanisms. One possible mechanism is that inhibition of Rpn4 degradation alters the expression of some NHEJ genes. For example, *YKU70* is downregulated in the RPN4* strain, and as a consequence, the recruitment of Yku70 to a DSB is reduced. Several other core (structural and/or catalytic) NHEJ genes, including *MRE11*, *YKU80*, and *NEJ1*, are also downregulated in the presence of Rpn4*. Interestingly, there is a 10-bp consensus sequence in the promoters of *YKU70*, *YKU80*, and *NEJ1* genes, suggesting that they may be coordinately regulated. We suspect that the effect of Rpn4* on the expression of these three genes may be indirect as no Rpn4 binding site is found in their promoters. It is possible that inhibition of Rpn4 degradation may lead to upregulation of a repressor, perhaps encoded by an Rpn4 target gene, which binds to the 10-bp consensus sequence and represses the NHEJ genes. In addition to the core NHEJ genes, the expression of *INO80*, which encodes a subunit of the INO80 chromatin remodeling complex that has been shown to play a role in NHEJ [44], [45], is downregulated in the RPN4* strain. This may also contribute to the NHEJ defect caused by inhibition of Rpn4 degradation. Interestingly, inhibition of Rpn4 degradation leads to upregulation of several NHEJ-related genes, including *RTT109*, which encodes a histone acetyltransferase that acetylates H3-K56 [51]. We found that overexpression of *RTT109* partially impaired NHEJ (data not shown). This observation is in contradiction to a previous report showing that Rtt109 is required for efficient NHEJ [43]. Perhaps, an appropriate expression level of Rtt109 is critical for NHEJ. Too much or too little Rtt109 would decrease the NHEJ efficiency.

Inhibition of Rpn4 degradation may affect NHEJ through another mechanism. We showed that Rpn4 is recruited to a DSB, suggesting that Rpn4 may directly participate in NHEJ. Interestingly, whereas their recruiting kinetics is similar, the release of Rpn4* from the DSB is markedly slower than that of Rpn4. This implies that release of Rpn4 from the DSB, perhaps through proteasomal degradation, may be important for NHEJ. It is worthy of note that proteasome dissociation from the DSB is concomitantly delayed with Rpn4* in the RPN4* strain. This observation suggests that Rpn4 and the proteasome may cooperate in NHEJ. Given that Rpn4 is a DNA-binding protein and a ligand and a substrate of the proteasome [4], [5], it is tempting to speculate that, on the one hand, Rpn4 assists in recruiting the proteasome to a DSB, and on the other hand, degradation of Rpn4 facilitates the release of the proteasome from the DSB. The role of the proteasome in NHEJ is likely to degrade one or more of the chromatin proteins surrounding the DSB to open up the space for the entrance of repair proteins. It is also possible that the proteasome degrades one or more of the repair proteins in the course of DSB repair. However, the proteasome may block the sequential recruitment of “late” repair proteins or interfere with the assembly of NHEJ complexes if its dissociation from the DNA lesion is delayed. Therefore, removal of the proteasome from a DSB can be critical for the proceeding of NHEJ. Whereas this model is attractive, we cannot definitely rule out the possibility that the prolonged association of Rpn4* on the DSB may be a result of the delay of the NHEJ process in the RPN4* strain. Further investigation is needed to address this possibility. We should point out that it remains possible that inhibition of Rpn4 degradation may also affect a post-NHEJ process and contribute to the reduced cell viability.

It is interesting to note that, while expression of Rpn4* clearly affects NHEJ, it displays no significant effect on HR. This observation suggests that proteasomal degradation of Rpn4 may be specifically required for the cell's commitment to NHEJ. Previous studies have shown that HR and NHEJ compete for DSBs; but the mechanism ruling this competition remains largely unclear [52], [53]. Alternatively, inhibition of Rpn4 degradation may not alter the expression of HR-related genes as sufficiently as it does on some of the NHEJ genes, and, therefore, does not affect HR. We are also aware of the possibility that the RPN4* cells may lose viability more quickly than wildtype cells in the presence of a persistent DSB. This difference is somewhat insignificant in the presence of HR, which manages to fix the continuously occurring DSB before the RPN4* cells lose viability. However, in the absence of HR, the persistent DSB may not be repaired in time by NHEJ, which is known less efficient than HR, leading to a lower survival rate of the RPN4* cells relative to their wildtype counterparts. This explanation reflects that inhibition of Rpn4 degradation not only impairs the NHEJ process, but may also make the cells more vulnerable to a persistent DSB.

Whereas the current study is focused on the biological relevance of Rpn4 degradation in DSB repair, we found that the RPN4* strain is hypersensitive to a variety of stressed conditions [28]. It will be of interest to investigate the functional role of proteasomal degradation of Rpn4 in other cellular pathways.

## Materials and Methods

### Yeast strains and plasmids

Yeast strains used in this study were listed in Table 1. Details of construction of the yeast strains and plasmids are available upon request. The replacement vector p305rpn4Δ::LEU2 for deletion of *RPN4* was previously described [3]. It carries 5′ and 3′ UTR fragments of *RPN4* linked by a Pst1 site in the pRS305 vector. To switch the selection marker from *LEU2* to *TRP1*, we digested p305rpn4Δ::LEU2 with Xho1 and Not1 and subcloned the insert into Xho1/Not1-cut pRS304 vector to obtain p304rpn4Δ::TRP1. Pst1-linearized p304rpn4Δ::TRP1 and p305rpn4Δ::LEU2 vectors were transformed into YFP17 to replace *RPN4*, resulting in YXY676 (*rpn4Δ::TRP1*) and YXY494 (*rpn4Δ::LEU2*) strains. Full-length *RPN4* and the truncated *RPN4_Δ1–10/Δ211–229_* allele (RPN4*, see ref. 28) were ligated with the *RPN4* promoter (∼500 bp) into the low-copy vectors pRS313 and pRS314 [54], resulting in p313RPN4, p314RPN4, p313RPN4* and p314RPN4* plasmids. For detection of the Rpn4 and Rpn4* proteins, a DNA sequence encoding a triple ha tag (3ha) was added right before the stop codon of *RPN4* and RPN4* in the p314RPN4 and p314RPN4* vectors to obtain p314RPN4-3ha and p314RPN4*-3ha. p314RPN4-3ha was cut with Spe1/Sac1 and the insert (C-terminal fragment of *RPN4* plus the 3ha tag) was subcloned into Spe1/Sac1-cut pRS306 vector to get p306RPN4_ΔN_-3ha. We then cut p306RPN4_ΔN_-3ha with Xho1/Sac1, and subcloned the insert into Xho1/Sac1-cut pRS304 vector to get p304RPN4_ΔN_-3ha. Bgl2-linearized p304RPN4_ΔN_-3ha vector was transformed into YFP17 to generate YXY506 (*RPN4-3ha::TRP1*) strain. p314RPN4* was digested with Xho1/EcoR1 and the 1.7 kb insert (500 bp *RPN4* promoter plus 1.2 kb N-terminal sequence of RPN4*) was subcloned into Xho1/EcoR1-cut pRS304 vector to get p304RPN4*ΔC. p314RPN4-3ha was cut with EcoR1/Sac1 and the insert was subcloned into EcoR1/Sac1-cut p304RPN4*ΔC vector to get p304RPN4*-3ha. Xho1-linearized p304RPN4*-3ha vector was transformed into YXY494 to get YXY512 (RPN4*-3ha::TRP1) strain. To generate yeast strains for ChIP analysis, we constructed a universal 3FLAG-tagged integration vector. Primers YX771 (TCGAC**GACTACAAGGACGACGATGACAAG**GGCGGAGCC**GATTATAAGGATGATGACGACAAG**GC, bolded are 2 FLAG repeats separated by a linker of Gly-Gly-Ala) and YX772 (GGCCGC**CTTGTCGTCATCATCCTTATAATC**GGCTCCGCC**CTTGTCATCGTCGTCCTTGTAGTC**G, bolded are 2 FLAG repeats separated by a linker of Gly-Gly-Ala) were annealed and subcloned into Sal1/Not1-cut 314CUPha-FLAG vector (7) to obtain 314CUPha-3FLAG. We then cut 314CUPha-3FLAG with Xho1/Sac1 and subcloned the insert into Xho1/Sac1-cut pRS306 vector to get the 306CUPha-3FLAG vector. We amplified a 770 bp C-terminal fragment of *YKU70* with primers YX779 (ACC*GGTACC*GTCAAGATCAGATAGTAATGG, italic is a Kpn1 site) and YX780 (CAA*GTCGAC*TATATTGAATTTCGGCTTTTT, italic is a Sal1 site). The PCR products were cut with Kpn1/Sal1 and subcloned into Kpn1/Sal1-cut 306CUPha-3xFLAG to obtain 306YKU70_ΔN_-3FLAG. Hind3-linearized 306YKU70_ΔN_-3FLAG vector was transformed into YX506 and YX512 to get YXY526 (a *YKU70-3FLAG::URA3* derivative of YXY506) and YXY532 (a *YKU70-3FLAG::URA3* derivative of YXY512). We amplified PRE6 ORF with primers YX239 (CAGTCTGCAGATGAGTGGTTACGATAGAGCT) and YX271 (GATC*CTCGAG*ATGGTTAGATTTTTTCTTTTTGTC, italic is an Xho1 site). The PCR products were cut with Kpn1/Xho1 and subcloned into Kpn1/Sal1-cut 306CUPha-3xFLAG to obtain 306PRE6_ΔN_-3FLAG. Bgl2-linearized 306PRE6_ΔN_-3FLAG vector was transformed into YX506 and YX512 to get YXY570 (a *PRE6-3FLAG::URA3* derivative of YXY506) and YXY572 (a *PRE6-3FLAG::URA3* derivative of YXY512). *RAD53* ORF without stop codon was amplified by PCR with primers YX766 (CAA*GAATTC*ATGGAAAATATTACACAACCC, italic is an EcoR1 site) and YX765 (ACCA*GCGGCCGCC*GAAAATTGCAAATTCTCGGG, italic is a Not1 site). The PCR products were digested with EcoR1/Not1 and subcloned into EcoR1/Not1-cut 314CUPha-FLAG vector to get 314CUPRAD53FLAG, which expresses C-terminally FLAG-tagged Rad53 from the *CUP1* promoter. All plasmids were verified by restriction enzyme analysis and DNA sequencing, and desired yeast strains were confirmed by PCR and immunoblotting analysis.

**Table 1:** *S. cerevisiae* strains used in this study.

Strains	Genotypes	Sources/References
EJY140	*MAT* **a** *trp1-Δ63 ura3-52 his3-Δ200 leu2-3,112 lys2-801 rpn4Δ::LEU2*	[3]
BY4741	*MAT* **a** *his3-Δ1 leu2Δ0 met15Δ0 ura3Δ0*	[56]
YGB655	*MAT* **a** *his3-Δ1 leu2Δ0 met15Δ0 ura3Δ0 sml1Δ::NatR*	A gift from G Brush
YGB656	*MAT* **a** *his3-Δ1 leu2Δ0 met15Δ0 ura3Δ0 sml1Δ::NatR mec1Δ::LEU2*	A gift from G Brush
*tof1Δ*	*MAT* **a** *his3-Δ1 leu2Δ0 met15Δ0 ura3Δ0 tof1Δ::KanR*	[56]
*tel1Δ*	*MAT* **a** *his3-Δ1 leu2Δ0 met15Δ0 ura3Δ0 tel1Δ::KanR*	[56]
YFP17	*hmlΔ::ADE1 mat* **a** *Δ::hisG hmrΔ::ADE1 leu2-cs ade3::GAL1::HO ade1 lys5 ura3-52 his4*	[39]
YXY494	an *rpn4Δ::LEU2* derivative of YFP17	This study
YXY506	an *RPN4-3ha::TRP1* derivative of YFP17	This study
YXY512	an RPN4*-3ha::TRP1 derivative of YXY494	This study
YXY526	a *YKU70-3FLAG::URA3* derivative of YXY506	This study
YXY532	a *YKU70-3FLAG::URA3* derivative of YXY512	This study
YXY570	a *PRE6-3FLAG::URA3* derivative of YXY506	This study
YXY572	a *PRE6-3FLAG::URA3* derivative of YXY512	This study
YXY676	an *rpn4Δ::TRP1* derivative of YFP17	This study

### Immunoblotting analysis

Yeast cells were grown to OD_600_ of 0.8–1.2, harvested and resuspended in lysis buffer (150 mM NaCl, 50 mM Tris.Cl, 5 mM EDTA, 1% Triton X-100, pH 7.5) plus protease inhibitor mix (Roche, Indianapolis). Yeast extracts were prepared using the glass-bead vortexing method [11]. Protein concentrations were measured by Bradford assay. Approximately 20 µg of each extract was resolved by SDS-PAGE, followed by immunoblotting with an anti-FLAG antibody for C-terminally FLAG-tagged Rad53, and detection with the Odyssey infrared imaging system (Li-Cor Biosciences). The blot was reprobed with an anti-yeast α tubulin antibody (Chemicon) to verify comparable loading.

### Analysis of gene expression

qRT-PCR was applied to measure gene expression. RNA was prepared from cells grown to OD_600_ of 0.8–1.2 as previously described [55]. Reverse-transcription was carried out with SuperScript II reverse transcriptase (Invitrogen), and qRT-PCR was performed using Fast SYBR Green Master Mix in the StepOne™ system following the manufacturer's instruction (Applied Biosystems). The *ACT1* gene served as an internal control for normalization of gene expression levels. The PCR primers for *ACT1* were YX814 (TCCATCCAAGCCGTTTTG) and YX815 (CGGCCAAATCGATTCTCA), whereas the primers used for NHEJ genes were listed in Table S1.

### Measurement of DSB induction and repair

Overnight cultures were diluted into raffinose medium and grown to OD_600_ of ∼0.8. Galactose was then added to 2% final concentration to induce HO endonuclease expression. For DSB induction analysis, cell samples were withdrawn at various time points after galactose induction. To measure DSB repair, galactose-induced cell cultures were transferred to glucose medium, and cells were collected at different time points after termination of HO expression. Genomic DNA was prepared from the cell samples and subjected to qRT-PCR analysis with a pair of primers (P1 and P2) flanking the HO cut site, which is inside a 117-bp fragment inserted at the *leu2* locus of YFP17 and its derivative strains [39]. P1 (TAGGTGCTGTGGGTGGTC) is 12 bp upstream from the insertion site whereas P2 (TTAGTAAACCTTGTTCAGGTC) is 8 bp downstream from the insertion site. Another pair of primers (YX718, CTCGGATCCAAGAAAAGGGAGCACCTG; YX719, CCTTCTAGAATCCTGTACACGGATGCC) amplifying an unrelated gene (*PRE1*) were used as control to normalize the qRT-PCR results.

### Chromatin immunoprecipitation assay

ChIP assay was carried out as described [27]. In brief, cell extracts were prepared after in vivo cross-linking of DNA and proteins with formaldehyde, and incubated with agarose beads conjugated with anti-FLAG antibody (Sigma) to precipitate Yku70-3FLAG or Pre6-3FLAG and bound DNA fragments. Or an anti-ha antibody (Covance) combined with Protein A agarose (Calbiochem) was used to bring down Rpn4-3ha or Rpn4*-3ha and bound DNA fragments. After the reversal of cross-links, DNA fragments were purified and analyzed by qRT-PCR with two sets of primers that amplified the segments ∼40 bp proximal or ∼100 bp distal to the DSB. The proximal primers are P3 (TGTCTGCCCCTAAGAAGATCG) and P4 (CAGCCTTCTTGGAGGCTTCCA) whereas the distal primers are P5 (AGACTTATCTCCAATCAAGCC) and P6 (GTGATTCTTTGCACTTCTGGA). The ChIP signals were calculated by normalizing the amount of PCR product from the immunoprecipitated DNA fragments to that from the input samples before immunoprecipitation, and expressed as relative ratios against the nonspecific ChIP signals at time zero (before HO induction) set at 1.0.

### Cell growth and survival assays

Cell growth was assessed by serial dilution assays. Yeast cells were grown to exponential phase and normalized by optical density. Five-fold serial dilutions of cells were spotted on selective synthetic complete (SC) plates and incubated at 30°C. Cell survival rates were measured by the colony formation assay. For MMS stress, overnight cultures were diluted and continued to grow to OD_600_ of 0.8∼1.2 before subjected to MMS treatment. Treated cells were extensively washed and seeded on selective SC plates. Untreated cells were spread on selective SC plates as control. For HR and NHEJ assays, cells were grown in pre-induction medium (raffinose medium) and then spread on plates containing either galactose or glucose. Colonies were counted 3–4 days after plating. Survival rates were measured as the ratios of colony counts after treatment to the counts without treatment, or the ratios of colony counts on galactose plates versus those on glucose plates. Each survival assay was repeated at least 3 times.

## Supporting Information

Table S1 (0.07 MB DOC)
