# Supplementary material for: Inhibition of Proteasomal Degradation of Rpn4 Impairs Nonhomologous End-Joining Repair of DNA Double-Strand Breaks
Source: PLoS One. 2010 Apr 1;5(4):e9877. doi: 10.1371/journal.pone.0009877 (PMC2848573; doi:10.1371/journal.pone.0009877)
Supplement: Table S1 — (0.07 MB DOC) [file pone.0009877.s001.doc]

**Table S1.** List of NHEJ genes analyzed by qRT-PCR and the corresponding primers

| Genes | Upstream primers | Downstream primers |
| --- | --- | --- |
| *PRE1* | YX732:CAAGAATTCATGGATATTATT CTGGGCATC | YX717: ACTGGATCCCCTAATTGA CTTGGCTAATTC |
| *RPT6* | YX126: CTGAGGGAAAATACATCGTGG | YX125: CAGGAACTTTTTCCACCATCA |
| *MRE11* | YX793: AAGAAATGATCCGCGACG | YX822: AACACGTCCCACAAATCG |
| *RAD50* | YX792: ATCATGGATTGAAAATGCAAG | YX824: CATCGTCCTCTCATATCC |
| *XRS2* | YX794: CCAGAATGGCATAAACGC | YX823: TCGTTTTCTTCTGTGCCC |
| *YKU70* | YX779: ACCGGTACCGTCAAGATCAGA TAGTAATGG | YX825: GGTTCTTATCGAACCTTC |
| *YKU80* | YX826: CCAGCAACATCTGTCGTT | YX827: CGTAGCAGGAAAAGCCTC |
| *DNL4* | YX795: GACGTTCGGTTAATCAGC | YX828: TTTTCAGCGACGGCTCTC |
| *LIF1* | YX829: AATGGACGTGCAGAATGC | YX830: CTCTCCTTTATATTCCGG |
| *NEJ1* | YX748: ACCGAATTCATGGATTCTGAG TTGAAAGGG | YX831: CTCTGGCATTGCTGTGAT |
| *RAD27* | YX804: CCAGAATTCATGGGTATTAAA GGTTTGAAT | YX832: TACCCATCAAGTGTGACG |
| *REV1* | YX833: TATCGAACTTTGGGCTGC | YX834: GAAAGCTCTGTTCGTCAC |
| *INO80* | YX835: TGTTTCAGCTCCTCCGGT | YX836: TCAGGGAATAGTCCTGTC |
| *VPS75* | YX837: ATGGCTGGCATTGGAGTC | YX838: GCCACTCAATTGGAACGG |
| *POL3* | YX839: TGACCAATATGAGGGTGC | YX840: GGTTGTGCGCCATCATAA |
| *POL4* | YX841: GTTCGTTCCGGTGAAGAA | YX842: TTCCGACCCAATGCCGTA |
| *RSC1* | YX843: TCTCTAGGTCGAATTCCG | YX844: AGAAGCAGAATGGGTTGG |
| *RSC2* | YX845: GTTGGTATTACAGACCCG | YX846: TTCGCAAACGAACAAGGG |
| *RSC3* | YX820: TATTGAGCGCAACAGTGG | YX821: GCTGATCATTGGTCACAC |
| *RSC4* | YX847: AGCAGGTGCGTTAGCTAT | YX848: ATAACCATTGCCGAGACC |
| *RSC6* | YX849: GATGAGGACAGTGCAGAG | YX850: TTTTCCACCGTCGACACC |
| *RSC8* | YX851: AGATGGAGCGGAACCACA | YX852: TGATTGACTCGTTACCGC |
| *RSC30* | YX853: AACTGACCAAACTAGGCG | YX854: CATCATGCATGTACAGAG |
| *YAF9* | YX855: TGAGATCCATAGAAGCGC | YX856: tcggaattcggtacagga |
| *DOA1* | YX857: GCCATCTCTATTTGGTCC | YX858: CACTTGCGTAGATAGTTC |
| *UBI4* | YX859: CGATACCATCGACAACGT | YX860: TGCATACCACCTCTTAGC |
| *FYV6* | YX861: GTGTCAGAAGGAGTTGGT | YX862: ACGGTCTCTCACCAAACC |
| *HTZ1* | YX863: AGGAAAAGCTCATGGAGG | YX864: TCCAGCACTTCAGCAGTC |
| *MCK1* | YX865: ATGCTTATCGGCAAAGCC | YX866: AACCTCTGTTGCGAAGAG |
| *ARD1* | YX867: ATTCGCAGAGCGACAATC | YX868: ATTGTCCTGCCGTCATTG |
| *APR6* | YX869: GCGCAGATCATAACACTG | YX870: TACCATAAGAGGTCGCAC |
| *SWC5* | YX871: ATGACGGTGTGAACAGAG | YX872: TGTAGCTCCTCCCAGATG |
| *BRE5* | YX873: CTGAATCGTCAACTCAGG | YX874: CTCTTCTGTAGAGACCTC |
| *MSN5* | YX875: TCTGGTATCCAACAGGAG | YX876: CAACTCGATACCACAAGC |
| *BUD31* | YX877: AGACCAGAAGATCCAAGC | YX878: CAGAGCTGCTCATTGGAC |
| *SRS2* | YX879: GCTACCGATGTGTCATGT | YX880: GTGCTCTGGAGCAACAAT |
| *CSE2* | YX881: TCTGCTGCCTACGAATCC | YX882: TCCTTATTTGGTGCAGCG |
| *CTI6* | YX883: AGACCGGAGTTACATCAG | YX884: GCTTCATTGGCGGCATGA |
| *LGE1* | YX885: ACTTGAGACAAACCGGAG | YX886: CCTGCCGTTCGTATCTTG |
| *APQ12* | YX887: ATGGACGCTACTCAACCG | YX888: ACCAACGTCAAATACAGG |
| *EMI5* | YX889: AGACACTGTCGTTGCAAG | YX890: CCAATGGCTCGTTAGTTC |
| *RRN9* | YX816: GGATAACTGGACGAGCTG | YX817: CATCATATCAGAGGCGTG |
| *RTT109* | YX818: TCCTGCTCGAGTAAGATC | YX819: TTCCTCTGCCAATTGGTG |
